# 

**Published:** 1843-11-25

**Authors:** 


### CONTENTS.

A Course of Clinical Lectures delivered at the Hotel Dieu, Paris, for the Session 1842–'3. By Dr. Chomel. Lecture 14, concluded, 265
Contributions to the History of Malaria. Communicated by Professor Dunglison, - - 268

*Bibliographical Notices.*

Practical Medicine; Illustrated by cases of the most Important Diseases. Edited by John M. Galt, M. D., - - - - 269
Anatomical Atlas, illustrative of the Structure of the Human Body. By Henry H. Smith, M. D., &c., - - - - - - - 269
The Dissector, or Practical and Surgical Anatomy. By Erasmus Wilson, M. D. - 270
Editorial, - - - - - - - 270

*Retrospect of the Medical Sciences.*

Dr. Ashwell on Irritable Uterus, - - - 270
On Bronchial Blenorrhœa. By Dr. Cheyne, 271
Extract of Indian Hemp, - - - - - 271
Vital Statistics of Sheffield, - - - 272
Sputa in Bronchitis. By Dr. Simon, - - 272
Formula for Opodeldoc, - - - - 273
The Hysteric Diathesis. By Dr. S. Ashwell, 273
Dr. Simon on the Urine in Pneumonia and Pleuro-Pneumonia, - - - - 273
Dr. Christison on the New Mode of Detecting Arsenic, - - - - - - - 274
Case of Dislocation of the Odontoid Process of the Axis, - - - - - - 274
Treatment or Cephalalgia, - - - 275
Un-united Fracture of the Leg Successfully Treated with Iodine, - - - - 275
Some Observations on the Statistics of the Vibrio Humana. By Dr. Knox. - - 275
Dr. Kennedy on Scarlatina, - - - - 276
Treatment of Hemicrania and Tic Douloureux. By M. Ducros, - - - - 276
Cream of Taraxacum, - - - - - 276
Lithotrity and Lithotomy in Children, - - 276

